# Supplementary material for: Asynchronous magnetic resonance elastography: Shear wave speed reconstruction using noise correlation of incoherent waves
Source: Magn Reson Med. 2022 Oct 27;89(3):990–1001. doi: 10.1002/mrm.29502 (PMC9792433; doi:10.1002/mrm.29502)
Supplement: Supplementary file 1 — DATA S1 MATLAB code used to generate the numerical simulations of Figure 1 [file MRM-89-990-s002.zip › k-Wave/helpfiles/findClosest.html]

findClosest :: Functions (k-Wave)


# findClosest

Return the closest value in a matrix.

## Syntax

```
value = findClosest(A, a)
[value, index] = findClosest(A, a)
```

## Description

`findClosest` returns the value and index of the item in A
that is closest to the value a. For vectors, value and index correspond to the closest element in A. For matrices, value and index are row vectors corresponding to the closest element from each column. For N-D arrays, the function finds the closest value along the first non-singleton dimension. If there is more than one element with the closest value, the index of the first one is returned.

## Inputs

|  |  |
| --- | --- |
| `A` | matrix to search |
| `a` | value to find |

## Outputs

|  |  |
| --- | --- |
| `value` | value in A that is closest to a |
| `index` | the index of a within A |

## See Also

`find`, `min`
